# Characterization of Integrons and Antimicrobial Resistance in *Escherichia coli* Sequence Type 131 Isolates

**DOI:** 10.1155/2020/3826186

**Published:** 2020-02-24

**Authors:** Jiangqing Huang, Fangjun Lan, Yanfang Lu, Bin Li

**Affiliations:** Department of Clinical Laboratory, Fujian Medical University Union Hospital, Fuzhou, Fujian 350001, China

## Abstract

**Background:**

*Escherichia coli* sequence type 131 (ST131) is an important multidrug-resistant extraintestinal pathogen, which can cause many kinds of infections. Integrons may play a crucial role in the dissemination of antibiotic resistance genes. The purpose of this study was to characterize the prevelance of integrons among *E. coli* ST131 strains in China.

**Methods:**

Eighty-three *E. coli* ST131 strains in China. *E. coli* ST131 strains in China.

**Results:**

Overall, 26.5% (22/83) of the *E. coli* ST131 strains in China. *dfrA17-aadA5* and *aac*(*6*′)*-Ib-cr-cmlA5*. Only one type of Pc promoter variant was detected among 22 integron-positive isolates (PcW). In vivo transfer of integron was successful for 9 of integron-positive *E. coli* ST131 strains in China. *E. coli* ST131 strains in China.

**Conclusions:**

Our study showed a low prevalence of integrons was detected in *E. coli* ST131. Continued surveillance of this mobile genetic element should be performed to study the evolution of antibiotic resistance among *E. coli* ST131.*E. coli* ST131 strains in China. *E. coli* ST131 strains in China.

## 1. Introduction

Integrons are genetic elements found in bacteria, which can capture and express antimicrobial resistance gene cassettes [1]. Integrons are strongly associated with the dissemination of antimicrobial genes among different genera of bacteria [2]. Several types of integron classes have been found nowadays in bacteria [3]. Integrons are often located on transmissible plasmids or transposons, which can facilitate the transferability of antimicrobial genes among bacteria [4].

*Escherichia coli* sequence type 131 (*E. coli* ST131) is an important multidrug-resistant extraintestinal pathogen. It is firstly identified in 2008 and now disseminated globally [5]. *E. coli* ST131 is becoming the predominant extraintestinal pathogenic *E. coli* lineage and can cause many clinical infections, such as sepsis and urinary tract infections [6]. *E. coli* ST131 are usually reported to produce extended spectrum *β*-lactamases and show resistance to fluoroquinones [7,8]. The emergence of such pathogen has made a significant contribution to the rising prevalence of antibiotic resistance [9].

Integrons, especially class 1 and 2 ones, have a worldwide distribution in *Enterobacteriaceae* [10, 11]. This has led to the speculation that integrons probably involve in the dissemination of antimicrobial resistance genes in *E. coli* ST131. Thus, in this study, we determined the distribution of various integrons among *E. coli* ST131 collected at a university hospital in southern China.

## 2. Materials and Methods

### 2.1. Bacterial Isolates

A total of 83 *E. coli* ST131 were used in this study, which were collected at a Chinese university hospital (Fujian Medical University Union Hospital, Fuzhou, Fujian province, China) between August 2014 and August 2015. These strains were isolated and reported in our previous study [8].

### 2.2. Antimicrobial Susceptibility Testing

The antimicrobial sensitivity was tested by the disk diffusion method according to the Clinical and Laboratory Standards Institute (CLSI, 2016) guidelines [12]. The antibiotics used for the susceptibility tests were cefotaxime (CTX), ceftazidime (CAZ), cefepime (FEP), ciprofloxacin (CIP), levofloxacin (LEV), amikacin (AMK), imipenem (IMP), piperacillin-tazobactam (TZP), aztreonam (ATM), trimethoprim-sulfamethoxazole (SXT), and ertapenem (ETP). *E. coli* ATCC 25922 was used for quality control.

### 2.3. Detection of Integrons, Their Pc Promoters, and Associated Resistance Genes

The presence of class 1, 2, and 3 integrons was analyzed by PCR in *E. coli* ST131 as described previously [13]. The characterization of the variable region of integrons was detected by PCR and DNA sequencing. In those isolates that were positive for *intI1*, the Pc promotor region was detected as described previously [14].

The presence of genes associated with ampicillin (*blaTEM*, *blaSHV*, and *blaOXA-1*), tetracycline (*tetA*-*tetE*), streptomycin (*aadA*), sulphonamide (*sul1*, *sul2*, and *sul3*), kanamycin (*aphA1* and *aphA2*), chloramphenicol resistance (*cmlA* and *floR*), and plasmid-borne quinolone resistance genes (*qnrA*, *qnrB*, and *qnrS*) was also analyzed by PCR [13, 15].

### 2.4. Conjugation Experiments and Plasmids Detection

A conjugation experiment was performed in broth culture with *E. coli J53 Az*^*r*^ as the recipient [16]. Conjugation experiments were carried out with integron-positive strains harboring resistance gene cassettes. Transconjugants were selected on MacConkey agar plates containing streptomycin at 50 mg/liter and sodium azide at 100 mg/liter for selection. The grown isolates were selected and identified by the Vitek system.

### 2.5. Pulsed-Field Gel Electrophoresis Analysis (PFGE)

The integron-positive *E. coli* ST131 isolates were subjected to molecular typing using XbaI digestion by pulsed-field gel electrophoresis, which clonally evaluates isolates. A PFGE dendrogram was constructed with BioNumerics software (Applied Maths, Sint-Martens-Latem, Belgium) according to the unweighted pair group method based on Dice coefficients. Isolates with a Dice similarity index ≥70% were considered to belong to the same PFGE cluster [17].

### 2.6. Statistical Analysis

SPSS22.0 statistical software was used for statistical analysis, and the chi-square test or Fisher's exact test (two tailed) or Mann–Whitney test was performed for data comparison. Only *p* < 0.05 was considered statistically significant.

## 3. Results

### 3.1. Integrons, Pc Promoters, and Associated Resistance Genes

Of the 83 *E. coli* ST131 isolates, 22 (27%) carried class 1 integrons. Class 2 and class 3 integrons were not detected in the study. The clinical characteristics of *E. coli* ST131 are showed in Table 1. The result of serotype revealed the integron-positive *E. coli* ST131 belonged to two types, and the most prevalence type was O25b (77.3%, 17/22). Results of phylogenetic analysis showed that 21/22 (95.5%) of isolates belonged to group B2 and 1/22 (4.5%) were D group. Several types of fragments ranging in size from 0.15 kb to 3.0 kb were identified (Table 2). Sixteen isolates carried a single integron with a same gene cassette (*dfrA17-aadA5*). Two gene cassette arrangements were detected in 19 (86.4%) of integron-positive isolates. In addition, only one isolate carried the *aac(6′)-Ib-cr-cmlA5* gene cassette array with the largest size (3.0 kb). In this study, only one Pc variant (PcW) was found in 22 integron-positive *E. coli* ST131, as shown in Table 1.

Table 3 shows the antimicrobial resistance genes detected in our *E. coli* ST131 isolates. The *blaTEM* gene was predominant in integron-positve *E. coli* ST131 isolates, and the *blaOXA-1* gene was in 9 isolates. The *blaSHV* gene was not found in this study. Regarding tetracycline resistance, *tetA* + *tetC* was more common in those strains with integron than those strains without integron. The following genes were detected among 56 SXT-resistant isolates (integron-positive/integron-negative isolates): *sul1* (15/7), *sul2* (2/5), and *sul1* + *sul2* (3/20). Among the 16 ciprofloxacin-resistant integron-positive isolates, the *qnrB* gene was detected in 2 isolates. Among the 22 integron-positive *E. coli* ST131 isolates, the *clmA* gene and *clmA* + *floR* gene were found in two different isolates. The *aadA* gene was observed in two of 22 integron-positive isolates, and none of these isolates carried *aphA1* and *aphA2* gene which conferred kanamycin resistance.

### 3.2. Antimicrobial Susceptibility Testing

*E. coli* ST131 showed high resistance to CTX (71.1%), CIP (69.9%), LEV (69.9%), and SXT (67.5%). However, resistance rates were low to ATM (36.1%), FEP (31.3%), CAZ (26.5%), AK (8.4%), IPM (3.6%), and TZP (1.2%). None of the isolates were resistant to ETP. The results of the antimicrobial susceptibility test were shown in Table 4. Compared with integron-negative *E.coli* ST131, integron-positive ones were shown to have a higher resistance to SXT and FEP (*p* < 0.05, respectively). Multidrug resistance (defined as resistance to six or more antibiotics) rates of integron-positive and -negative *E.coli* ST131 isolates were 45.5% and 18.0%, respectively.

### 3.3. Conjugation Experiments

In this study, transfer of integrons was successful in 9 of 17 integron-positive *E. coli* ST131 isolates harboring resistance gene cassettes (Table 1). The presence of integrons in the transconjugants was confirmed by PCR.

### 3.4. Bacterial Clonal Relatedness

**Figure 1:**
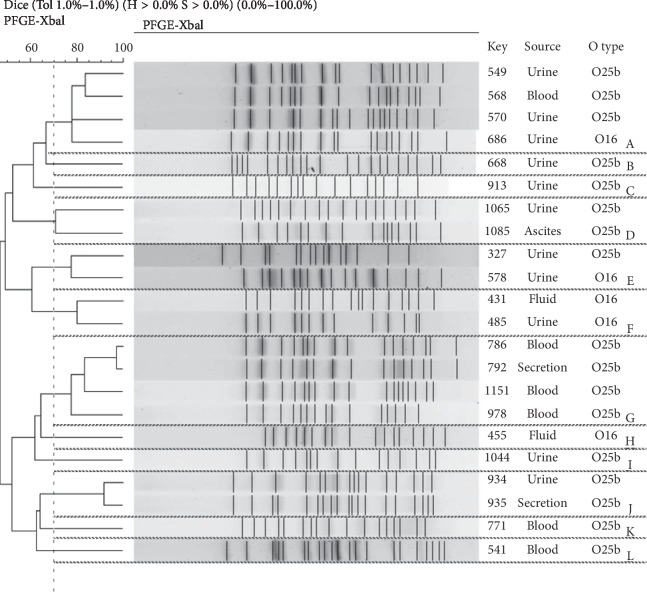
Dendrogram generated by bionumerics software that demonstrates the relationship of 22 integron-positive *E. coli* ST131 isolates at 70% similarity level, after digestion with *XbaI*. Strain designation, sample source, and O serotype are shown. The analysis of the bands generated was performed using the Dice coefficient and unweighted pair group method.

PFGE analysis of 22 integron-positive *E. coli* ST131 isolates revealed that these isolates were divided into 12 clusters (named A-L, in Figure 1) using 70% similarity cutoff value. Two clusters (A and G) were frequent, containing 4 isolates, respectively.

PFGE analysis of the 83 *E. coli* ST131 isolates (integron-positive and -negative isolates) demonstrated these subtypes were grouped into 22 different PFGE clusters (named 1–22, in Supplementary Data) using 70% similarity cutoff value. The integron-positive isolates were scattered in 10 groups. And 10/22 (45.5%) of integron-positive isolates were clustered into the same clonal group (3).

## 4. Discussion

This study demonstrated the occurrence of integrons among *E. coli* ST131 clinical isolates. The results showed that the prevalence of integrons was low, and some were located on transferable plasmids. The prevalence of the integron (26.5%) in this study was similar to that in our previous report (26.7%) [18]; however, it is lower than that found in clinical *Enterobacteriaceae* isolates in China (59.9%) [19]. The result suggested that the prevalence of integron was lower in *E. coli* ST131 as compared with other clinical isolates.

*E. coli* ST131 isolates showed high antimicrobial resistance to SXT, CIP, LEV, and CTX. In China, sulfonamides (such as SXT), *β*-lactams (such as CTX), and quinolones (such as CIP) are often used to cure *E. coli* infections; therefore, the high resistance of *E. coli* ST131 may be due to the frequent and inappropriate use of these antimicrobials [20–22].

Integrons are known to be correlated with multidrug resistance (MDR) [23, 24]. Multiple antibiotic resistance rates of integron-positive and -negative strains were 45.5% and 13.3%, respectively. This survey demonstrated MDR are widely distributed in *E. coli* ST131 (Table 1).

As previous studies reported, many antibiotic resistance gene cassettes in integrons probably play an important role in the development of antibiotics resistance [2, 25]. In this study, integrons were significantly associated with SXT. Two different gene cassettes were detected (Table 2), which correlated with resistance to a variety of aminoglycosides (*aac* and *aad*), chloramphenicol (*cmlA*), and trimethoprim (*dfrA17*), and the *dfrA17-aadA5* (84.2%) gene cassette dominated in integron-positive isolates in this study, which may contributed to resistance to SXT. The gene cassettes identified were not novel in this study, and it was also the most prevalent gene cassette in *E. coli* in other previous studies [26, 27]. According to the database in GenBank, a novel cassette array, *aac(6′)-Ib-cr-cmlA5*, was first reported in *E. coli*, suggesting the ability of integrons to capture and integrate resistance gene and to form novel cassettes that mediate resistance and multidrug resistance of *E. coli* ST131 clinical isolates. However, the *aac(6′)-Ib-cr-cmlA5*-carrying isolate was found to be susceptible (or intermediate) to all antimicrobials (Table 1) which indicated that there may exist some unknown mechanism leading to this phenomenon.

We also explored the presence of antimicrobial resistance genes that played a significant role in antimicrobial resistance (Table 3). The *bla*_*OXA-1*_ gene was found in 9 of 22 integron-positive isolates. This gene was often found as gene cassettes while not detected in variable region in this study [28, 29]. The fact could be explained by the limitation of PCR or loss of 5′ and 3′ conserved segments of integrons. Regarding *tet* gene, the *tetA* gene was the most common one in integron-positive isolates, supporting the previous reports highlighting that the *tetA* gene was located on the same conjugative plasmid with integron [30]. In this study, the higher percentage of resistance to STX might be attributed to the higher prevalence of *sul* gene among those integron-positive isolates, and this result corroborates a study reported previously that *sul1* gene is usually encoded by class 1 integrons and is part of the 3′ conserved segment [31].

Additionally, similar to another previous study, PcW was the most frequent common promoter in clinical *E. coli* isolates in this study, a relatively weak promoter [32]. However, compared with another previous finding in other bacteria, only one promoter was detected in this study that was a strange phenomenon [33, 34]. As discussed before, the prevalence of the promoter in *E. coli* ST131 and whether this is a feature of *E. coli* ST131 strains should be further investigated. Furthermore, the weaker Pc promoter showed the more efficient excision activity, which promotes integration of exogenous gene cassettes, and these suggested that PcW-carrying *E. coli* ST131 isolates have a strong capability to excise and capture gene cassettes, that might promote the spread of antimicrobial resistance [35].

In this study, 9 (52.9%) *E. coli* ST131 isolates transferred integron and resistance gene cassettes to *E. coli J53 Az*^*r*^ by conjugation. Many previous studies found that most of the resistance determination and integron in *E. coli* were encoded in a transferable plasmid, which might be horizontally transferred to the isolates among *E. coli* ST131 or different bacteria species by conjugation, potentially resulting in multiple drug resistance of bacteria [36, 37].

The result of PFGE demonstrated that integron-positive isolates were highly diverse in this study and there was no dominant clone (Figure 1). Interestingly, nearly half of integron-positive isoaltes belonged to the same group when we performed PFGE analysis on all isolates (Supplementary Data). Future study will be carried out to investigate this phenomenon.

## 5. Conclusions

In conclusion, a low prevalence of integrons was detected in *E. coli* ST131 clinical isolates. Continued surveillance of this mobile genetic element should be performed to study the evolution of antibiotic resistance among *E. coli* ST131.

## Figures and Tables

**Table 1:** Phenotypic and genotypic characteristics of 22 integron-positive *E. coli* ST131 isolates.

Isolates	Ward	Source	Age	Sex	Genetic material in isolate with integron		Serotype	Phylogenetic group	Resistance proflies	Conjugation experiments
					Promoter	Gene cassette(s)				
EC327	Urinary surgery	Urine	48	F	PcW	−^1^	O25b	B2	SXT	

EC431	Emergency surgery	Drain	78	F	PcW	−	O16	B2	CTX, CAZ, FEP, CI P, LEV, ATM, SXT CTX, CIP, LEV, SXT

EC455	Colorectal surgery	Drain	62	F	PcW	*dfrA17-aadA5*	O16	B2		+^2^

EC485	Urinary surgery	Urine	60	M	PcW	*aac(6′)-Ib-cr-cmlA5*	O16	B2	−	

EC541	Neurosurgery	Blood	69	M	PcW	*dfrA17-aadA5*	O25b	B2	CTX, SXT	

EC549	Neurosurgery	Urine	39	F	PcW	*dfrA17-aadA5*	O25b	B2	CTX, CAZ, FEP, CIP, LEV, AMK, ATM, SXT	

EC568	Hepatobiliary surgery	Blood	75	M	PcW	*dfrA17-aadA5*	O25b	B2	CTX, CAZ, FEP, CIP, LEV, AMK, ATM, SXT	

EC570	Urinary surgery	Urine	51	F	PcW	*dfrA17-aadA5*	O25b	B2	CTX, FEP, CIP, LEV, AMK, ATM, SXT	

EC578	Urinary surgery	Urine	68	M	PcW	*dfrA17-aadA5*	O16	D	CTX, FEP, CIP, LEV, SXT	+

EC668	Oncology	Urine	59	F	PcW	*dfrA17-aadA5*	O25b	B2	CTX, CIP, LEV, SXT	+

EC686	Cardiology	Urine	78	M	PcW	*dfrA17-aadA5*	O16	B2	CIP, LEV, SXT	+

EC771	Outpatient	Blood	61	F	PcW	-	O25b	B2	SXT	

EC786	Nephrology	Blood	64	F	PcW	*dfrA17-aadA5*	O25b	B2	CTX, CAZ, FEP, CI P, LEV, ATM, SXT	+

EC792	Colorectal surgery	Secretion	52	M	PcW	*dfrA17-aadA5*	O25b	B2	CTX, CAZ, FEP, CIP, LEV, ATM, SXT	

EC913	Gastrointestinal surgery	Urine	52	F	PcW	*dfrA17-aadA5*	O25b	B2	CTX, CIP, LEV, SXT	+

EC934	Hematology	Urine	54	F	PcW	−	O25b	B2	SXT	

EC935	Emergency surgery	Secretion	55	F	PcW	−	O25b	B2	SXT	

EC978	Hematology	Blood	43	F	PcW	*dfrA17-aadA5*	O25b	B2	CTX, CAZ, FEP, CI P, LEV, ATM, SXT	+

EC1044	Hematology	Urine	64	F	PcW	*dfrA17-aadA5*	O25b	B2	CTX, CAZ, FEP, CI P, LEV, ATM, SXT	+

EC1065	Urinary surgery	Urine	56	M	PcW	*dfrA17-aadA5*	O25b	B2	SXT	

EC1085	Oncology	Ascitic fluid	64	F	PcW	*dfrA17-aadA5*	O25b	B2	CTX, CAZ, FEP, CI P, LEV, ATM, SXT	+

EC1151	Outpatient	Blood	82	M	PcW	*dfrA17-aadA5*	O25b	B2	CTX, FEP, CIP, LEV, ATM, SXT	

F, female; M, male; CTX, cefotaxime; CAZ, ceftazidime; FEP, cefepime; CIP, ciprofloxacin; LEV, levofloxacin; AK, amikacin; IPM, imipenem; TZP, piperacillin-tazobartan; ATM, aztreonam; SXT, trimethoprim-sulfamethoxazole; ETP, ertapenem, ^1−^: not detected, ^2+^: successfully transferred.

**Table 2:** Size, number, and gene cassetes amplified from the variable region of integron in *E.coli* ST131.

Approximate length (kb)	Isolate (*n*)	Gene cassette (*s*)
0.15^1^	2	—
1.6	4	*dfrA17-aadA5*
1.6 + 0.7^2^	12	*dfrA17-aadA5*
3.0	1	*aac* (*6*′)*-Ib-cr-cmlA5*
Total	19	

^1^The 0.15 kb amplicon was found to be a integron variable region without any gene cassette present but contained partial sequences of 5′ and 3′ conserved segments of integrons. ^2^The 0.7 kb amplicon was found to be a integron variable region without any gene cassette present.

**Table 3:** Genes of resistance detected among *E.coli* ST131 strains with and without integrons.

Antibiotics	Integron-positive strains (*n* = 22)		Integron-negative strains (*n* = 61)	
	No. of resistance isolates	Genes detected (no. of strains)	No. of resistance isolates	Genes detected (no. of strains)
Ampicillin	—^*∗*^	*bla*_*TEM*_ (11)	—	*bla*_*TEM*_ (23)
		*bla*_*OXA-1*_ (9)		*bla*_*OXA-1*_ (1)
Tetracycline	—	*tetA* (1)	—	*tetA* (20)
		*tetC* (3)		*tetB* (2)
		*tetA* + *tetB* (6)		*tetC* (8)
		*tetA* + *tetC*(10)		*TetA* *+* *tetC* (10)
				*tetB* *+* *tetC* (1)
Sulphonamide	21	*sul1* (15)	35	*sul1* (7)
		*sul2* (3)		*sul2* (5)
		*sul1 + sul2* (3)		*sul1 + sul2* (20)
Ciprofloxaci	16	*qnrB* (2)	43	*qnrS* (1)
			
Streptomycin	—	*aadA* (2)	—	ND
Kanamycin	—	ND	—	*aphA1* (1)
				*aphA1 + aphA2* (1)
Chloramphenicol	—	*cmlA* (1)	—	*floR* (2)
		*clmA* *+* *floR* (1)		

^*∗*^Not detected; ND:no gene detected

**Table 4:** Association between antibiotic profile and integron in *E. coli* ST131.

Antibiotic	Antibiotic susceptibility									*p* value
	All isolates (*n* *=* 83)			Integron-positive isolates (*n* *=* 22)			Integron-negative isolates (*n* = 61)			
	*S* (%)	*I* (%)	*R* (%)	*S* (%)	*I* (%)	*R* (%)	*S* (%)	*I* (%)	*R* (%)	
CTX	25.3	3.6	71.1	31.8	0.0	68.2	23.0	4.9	72.1	0.726
CAZ	49.4	24.1	26.5	54.5	9.1	36.4	47.5	29.5	23.0	0.222
FEP	55.4	13.3	31.3	50.0	0.0	50.0	57.4	18.0	24.6	0.028
CIP	28.9	1.2	69.9	27.3	4.5	68.2	29.5	0.0	70.5	0.840
LEV	30.1	0.0	69.9	31.8	0.0	68.2	29.5	0.0	70.5	0.840
AK	79.5	12.1	8.4	68.2	18.2	13.6	83.6	9.8	6.6	0.564
IPM	96.4	0.0	3.6	100.0	0.0	0.0	95.1	0.0	4.9	0.562
TZP	95.2	3.6	1.2	90.9	9.1	0.0	96.8	1.6	1.6	1.000
ATM	37.4	26.5	36.1	50.0	4.5	45.5	32.8	34.4	32.8	0.289
SXT	32.5	0.0	67.5	4.5	0.0	95.5	42.6	0.0	57.4	0.003
ETP	98.8	1.2	0.0	100.0	0.0	0.0	98.4	1.6	0.0	—

CTX, cefotaxime; CAZ, ceftazidime; FEP, cefepime; CIP, ciprofloxacin; LEV, levofloxacin; AK, amikacin; IPM, imipenem; TZP, piperacillin—tazobartan; ATM, aztreonam; SXT, trimethoprim-sulfamethoxazole; ETP, ertapenem.

## Data Availability

The data used to support the findings of this study are available from the corresponding author upon request.

## Supplementary Materials

Supplementary Materials Pulsed-field gel electrophoresis (PFGE) of XbaI-digested DNA from 83 ST131 *E. coli* isolates. Red box showed the 22 integron-positive ST131 *E. coli* isolates at a 70% similarity level. Supplementary Materials
