# Supplementary figures and images for: Characterization of Integrons and Antimicrobial Resistance in Escherichia coli Sequence Type 131 Isolates
Source: Can J Infect Dis Med Microbiol. 2020 Feb 24;2020:3826186. doi: 10.1155/2020/3826186 (PMC7060437; doi:10.1155/2020/3826186)

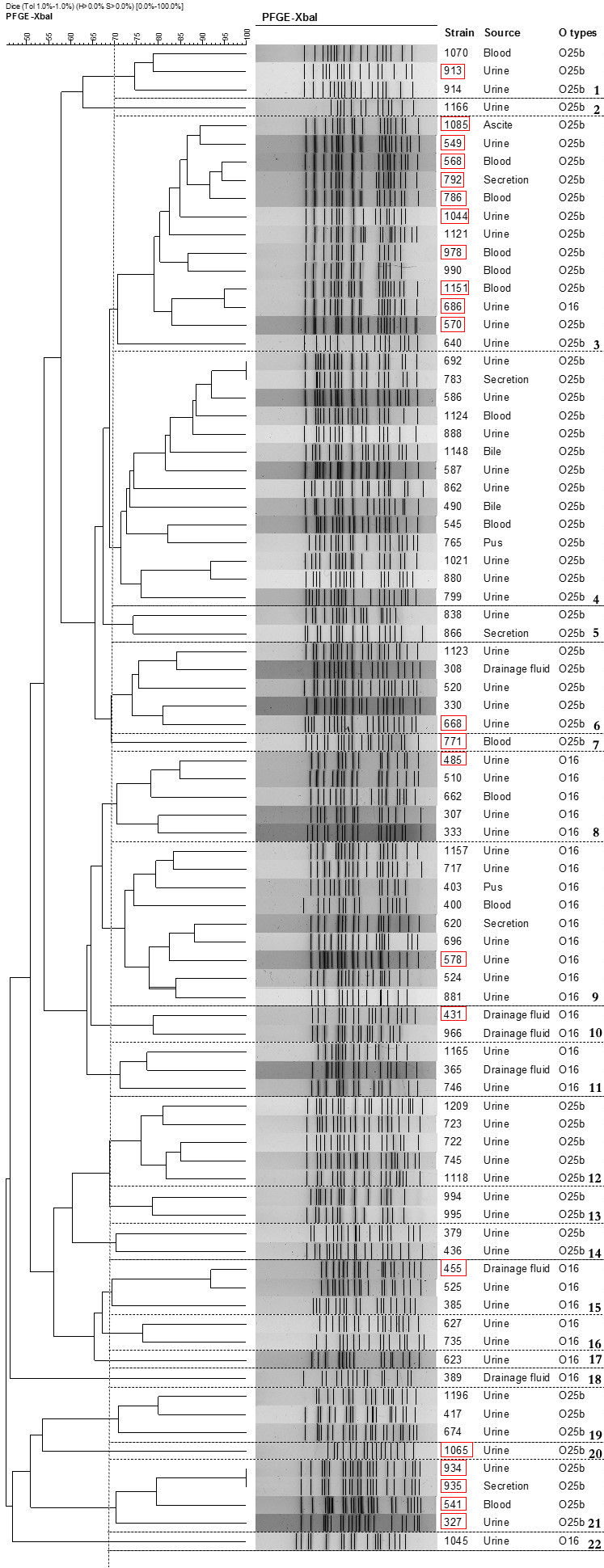

Supplement: Supplementary Materials — Pulsed-field gel electrophoresis (PFGE) of XbaI-digested DNA from 83 ST131 E. coli isolates. Red box showed the 22 integron-positive ST131 E. coli isolates at a 70% similarity level. Supplementary Materials [file 3826186.f1.docx]
